# Genome-Wide CRISPR-Cas9 Screen Does Not Identify Host Factors Modulating Streptococcus agalactiae β-Hemolysin/Cytolysin-Induced Cell Death

**DOI:** 10.1128/spectrum.02186-21

**Published:** 2022-02-02

**Authors:** Ifrah Shahi, Cristina N. Llaneras, Sofya S. Perelman, Victor J. Torres, Adam J. Ratner

**Affiliations:** a Department of Microbiology, New York University Grossman School of Medicine, New York, New York, USA; b Antimicrobial-Resistant Pathogens Program, New York University Grossman School of Medicine, New York, New York, USA; c Department of Pediatrics, New York University Grossman School of Medicine, New York, New York, USA; Ohio State University

**Keywords:** cytolysin, group B *Streptococcus*, intermedilysin

## Abstract

Pore-forming toxins (PFTs) are commonly produced by pathogenic bacteria, and understanding them is key to the development of virulence-targeted therapies. Streptococcus agalactiae, or group B Streptococcus (GBS), produces several factors that enhance its pathogenicity, including the PFT β-hemolysin/cytolysin (βhc). Little is understood about the cellular factors involved in βhc pore formation. We conducted a whole-genome CRISPR-Cas9 forward genetic screen to identify host genes that might contribute to βhc pore formation and cell death. While the screen identified the established receptor, CD59, in control experiments using the toxin intermedilysin (ILY), no clear candidate genes were identified that were required for βhc-mediated lethality. Of the top targets from the screen, two genes involved in membrane remodeling and repair represented candidates that might modulate the kinetics of βhc-induced cell death. Upon attempted validation of the results using monoclonal cell lines with targeted disruption of these genes, no effect on βhc-mediated cell lysis was observed. The CRISPR-Cas9 screen results are consistent with the hypothesis that βhc does not require a single nonessential host factor to mediate target cell death.

**IMPORTANCE** CRISPR-Cas9 forward genetic screens have been used to identify host cell targets required by bacterial toxins. They have been used successfully to both verify known targets and elucidate novel host factors required by toxins. Here, we show that this approach fails to identify host factors required for cell death due to βhc, a toxin required for GBS virulence. These data suggest that βhc may not require a host cell receptor for toxin function or may require a host receptor that is an essential gene and would not be identified using this screening strategy.

## INTRODUCTION

Streptococcus agalactiae, or group B Streptococcus (GBS), is a Gram-positive, beta-hemolytic bacterium and opportunistic pathogen that colonizes the rectovaginal tracts of 18% of pregnant women worldwide (1). GBS has frequently been associated with preterm births (2), stillbirths (3, 4), and severe neonatal infections, including sepsis and meningitis (5–8). Furthermore, the incidence of invasive GBS disease in nonpregnant adults has increased in recent years, particularly among the elderly and the immunocompromised (9, 10).

A major virulence factor employed by GBS during pathogenesis is the pore-forming toxin (PFT) known as β-hemolysin/cytolysin (βhc). In GBS, hemolytic activity, which is mediated by βhc, is tightly linked with pigment production. Recent data are consistent with the hypothesis that the ornithine rhamnolipid pigment (also called granadaene) and βhc are the same molecule (7). The structure of granadaene has been determined, and it consists of a polyene chain flanked by polar head groups on either end (11). Hyperhemolytic and hyperpigmented strains of GBS are frequently associated with invasive infections; in contrast, nonhemolytic strains of GBS are generally nonpigmented and less virulent (7, 12–14).

βhc plays a role in promoting host inflammatory responses and contributes to decreased barrier resistance in the lungs (15, 16) and brain (8), as well as at the maternal-fetal interface (7, 13). Purified βhc is cytotoxic to a wide range of eukaryotic cell types, including human and sheep erythrocytes (7), murine fibroblasts (17), and human immune cells, such as macrophages (12), neutrophils (18), and mast cells. Little is known about the mechanism by which βhc targets host cells, in part due to the limited solubility and unstable nature of βhc when purified. βhc forms transient pores of various sizes (19), and preincubation of βhc with phospholipids inhibits its hemolytic activity (17, 20). Experimentation with synthetic βhc analogs has demonstrated that the polyene chain length and the polar head groups affect the hemolytic potency of βhc (21). However, the identity of specific host factors required for βhc-mediated cell death remains unknown.

Recent advances in CRISPR-Cas9-based technologies have made it possible to create host cell libraries that can be screened for host genes required for susceptibility to bacterial toxins. This strategy has been used successfully to uncover novel host factors implicated in toxin-mediated lysis, such as sphingomyelin synthase 1 playing a role in susceptibility to Staphylococcus aureus alpha-toxin (22), the frizzle family (FZD) of receptors for Wnt signaling mediating Clostridioides difficile toxin B-induced cell rounding and death (23), HVCN-1 for the S. aureus LukAB toxin (24), and two genes (TM9SF2 and LAPTM4A) of previously unknown function mediating cytotoxicity induced by enterohemorrhagic Escherichia coli type III secretion systems and Shiga toxins (25). In this study, we used a CRISPR-Cas9 library targeting 19,050 human genes to screen for host factors required for βhc toxicity in HeLa cells. As a control, we conducted in parallel a CRISPR-Cas9 screen with the cholesterol-dependent cytolysin intermedilysin (ILY), which has a previously identified host cell surface receptor (26, 27). While the screening approach identified the ILY receptor CD59, as well as other factors affecting CD59 expression on the cell surface, none of the targets identified for βhc susceptibility could be validated by postscreen experiments. Based on these results, we conclude that while the CRISPR-Cas9 screening system is efficient and effective, it did not identify specific factors required for βhc-mediated cytotoxicity. These data are consistent with the hypothesis that no single nonessential receptor for βhc is present on the surface of HeLa cells.

## RESULTS

### CRISPR-Cas9 screen.

**FIG 1:**
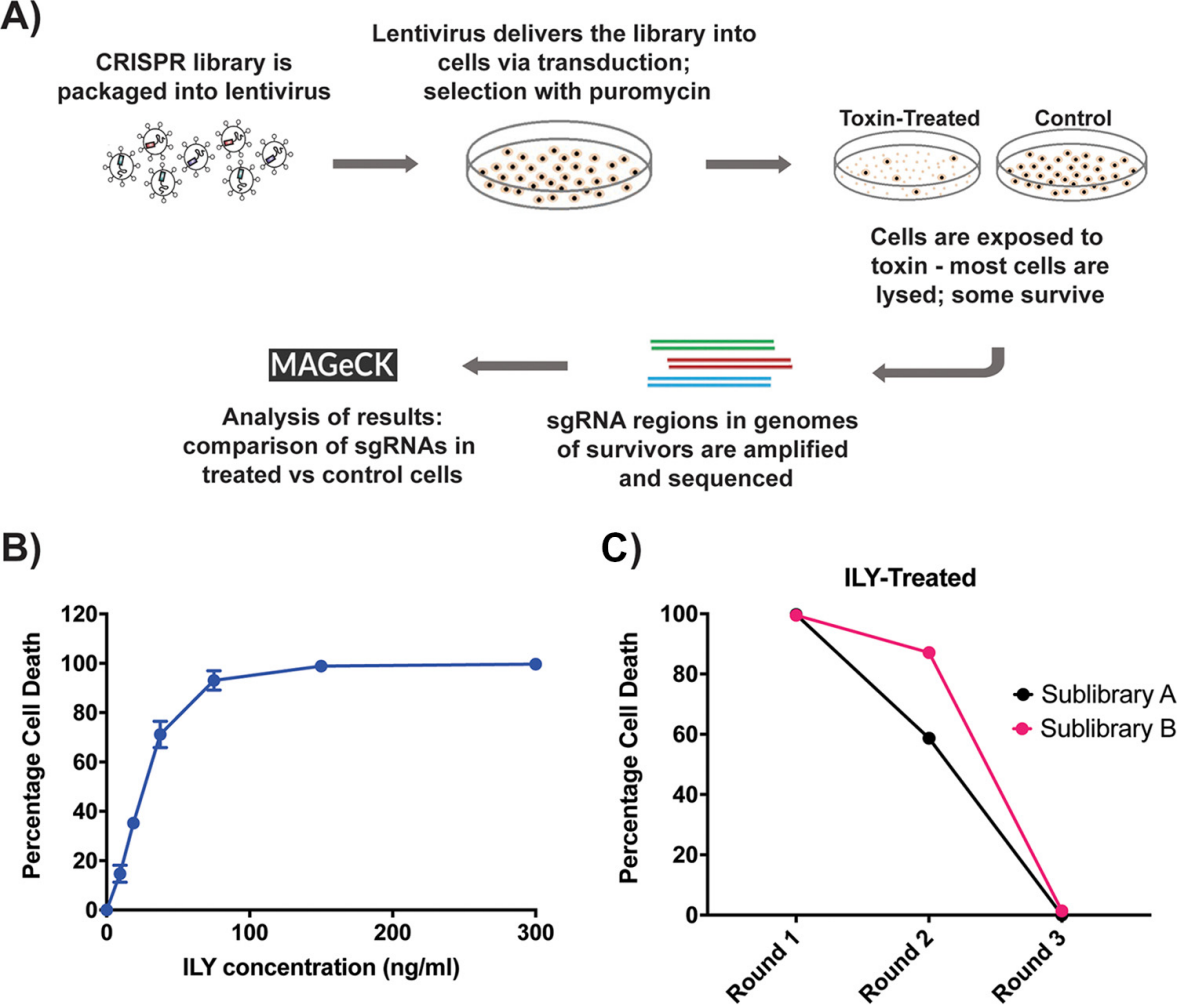
Conducting CRISPR-Cas9 knockout screens in HeLa cells with ILY. (A) Schematic representation of the CRISPR-Cas9 knockout screen. The plasmid library was packaged into lentivirus, and the population of HeLa cells transduced with the lentiviral vectors. Positively transduced cells were selected with puromycin before being exposed to two rounds of selection with toxin. DNA was extracted from the treated and control cell populations, the guide sequences amplified with PCR, and the PCR products sequenced via high-throughput sequencing analysis. Analysis of the results was conducted using MaGeCK software. (B) Percentages of cell death in HeLa cells when exposed to increasing concentrations of the ILY. Based on these results, 100 ng/ml of ILY was used for the CRISPR-Cas9 screen. The results shown are from one representative assay each of >2 repeats. Each point is the mean value from 3 replicates, and error bars represent ±SD. Some error bars are contained within the point and therefore not visible. (C) Percentages of cell death in the two CRISPR sublibraries of HeLa cells after each round of toxin exposure as part of the CRISPR-Cas9 screen. The CRISPR sublibraries of cells underwent three rounds of toxin exposure, and the percentages of cell death decreased with each round of ILY treatment.

We chose HeLa cells, a cervical cancer cell line, for our CRISPR-Cas9 screens. The screens were conducted with a genome-wide single guide RNA (sgRNA) library (Genome-scale CRISPR Knock-Out [GeCKO] version 2 [v2]) targeting 19,050 human genes (28, 29). This library includes six distinct sgRNAs targeting each gene, in addition to 1,000 control sgRNAs designed to have minimal homology with human genome sequences. The GeCKO v2 library was transduced into HeLa cells using lentiviruses (Fig. 1A) at a multiplicity of infection (MOI) of 0.3, to ensure that each cell only received a single sgRNA. Coverage of 500× (i.e., the number of cells was 500 times the number of sgRNAs present in the library) was maintained during transduction (Fig. 1A).

**FIG 2:**
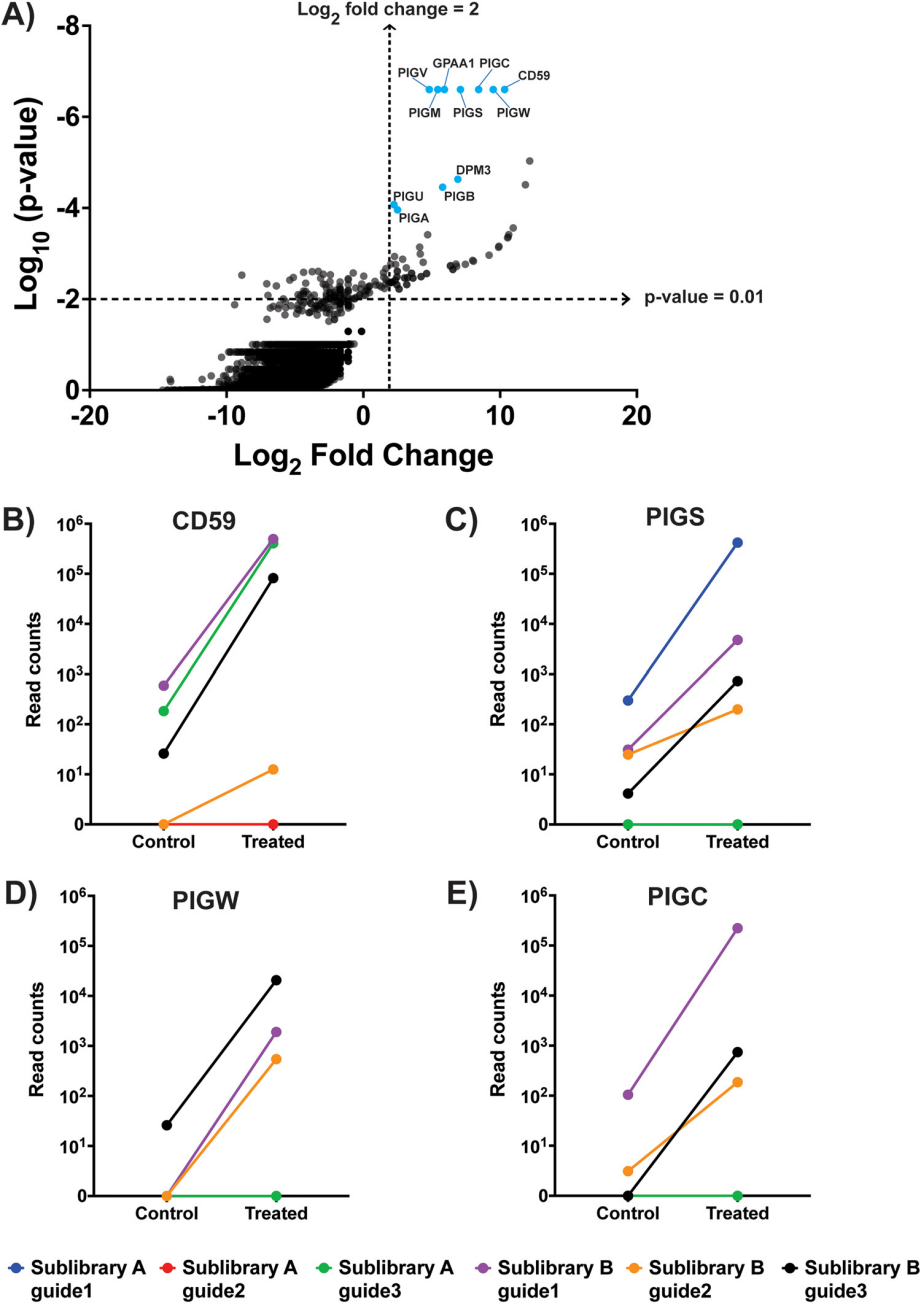
Significant hits from the CRISPR-Cas9 screen with ILY. (A) Results of the CRISPR-Cas9 knockout screen with ILY. The *x* axis represents log fold change averaged for all guides against each gene in the treated population of cells versus the control population of cells. The labeled genes, colored blue, are those considered significant based on a log fold change of ≥2, a *P* value of <0.01, ≥2 sgRNAs enriched in the ILY-treated versus control cell populations, and a false discovery rate (FDR) of <20%. (B to E) Read counts of all guides for the top 4 gene hits, as measured in the ILY-treated and the control cell populations. An increase in read count in the ILY-treated cells compared to that in the control cells indicates enrichment of that guide RNA through the course of the screening process.

As a control screen, transduced cells were subjected to three rounds of exposure to recombinant ILY at 100 ng/ml, a concentration that caused ≥90% cell death in wild-type HeLa cells (Fig. 1B). We chose to conduct three rounds of intoxication in order to increase the stringency of selection, weed out false positives, and ensure that only fully resistant clones survive. We observed that the percentage of cell death declined with each round of toxin exposure, consistent with positive selection of resistant clones (Fig. 1C). At the end of the third round, DNA was extracted from the surviving cells, followed by nested PCR amplification and next-generation sequencing (NGS) of the integrated sgRNA sequences. The sequencing results were analyzed using the software MaGeCK, which analyzes the distribution of sgRNAs across the treatment and control samples using a negative binomial (NB) model. MaGeCK then calculates *P* values for the sgRNAs using the NB model, ranks the sgRNAs based on these *P* values, and identifies positively or negatively selected genes using a robust ranking aggregation algorithm named α-RRA (30). From the MaGeCK rankings of the top enriched sgRNA targets for ILY-mediated cell death, we considered the most likely targets to be those with a false discovery rate (FDR) of ≤0.2, a *P* value of ≤0.01, a log_2_ fold change of ≥2 in the toxin-treated cell population relative to the control cell population, and at least 2 (of a possible 6) enriched sgRNAs (Fig. 2).

The top-ranked gene target in the ILY screen list was CD59, a glycosylphosphatidylinositol (GPI)-anchored protein known to be a required cell surface receptor for ILY-mediated pore formation (26, 27). Additionally, the next 14 top-ranked genes after CD59 were all involved in the GPI anchor biosynthesis pathway (Fig. 2). These results, collectively, indicate that the CRISPR-Cas9 screening strategy was successful at identifying (i) the known cell surface receptor of ILY (CD59), as well as (ii) individual components of GPI anchor synthesis and trafficking (such as PIGS, PIGW, and PIGC), which affect the cell surface availability of CD59 (Fig. 2B to E).

**FIG 3:**
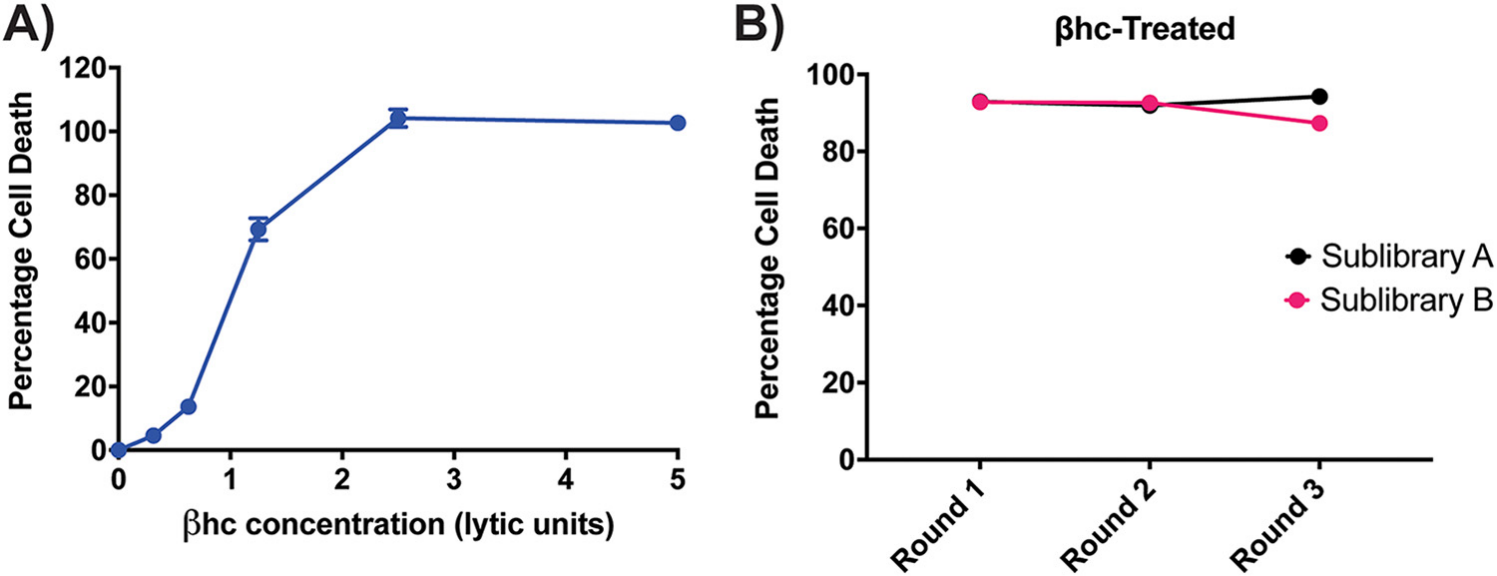
Conducting CRISPR-Cas9 knockout screens in HeLa cells with βhc. (A) Percentages of cell death in HeLa cells when exposed to increasing concentrations of βhc. Based on these results, 3 lytic units of βhc were used for the CRISPR-Cas9 screen. One lytic unit is the concentration needed for 50% HeLa cell lysis. The results shown are from one representative assay each of >2 repeats. Each point is the mean value from 3 replicates, and error bars represent ±SD. Some error bars are contained within the point and therefore not visible. (B) Percentages of cell death in the two CRISPR sublibraries of HeLa cells after each round of toxin exposure as part of the CRISPR-Cas9 screen. The CRISPR sublibraries of cells underwent three rounds of toxin exposure, and the percentages of cell death was unchanged over the three rounds of βhc treatment.

**FIG 4:**
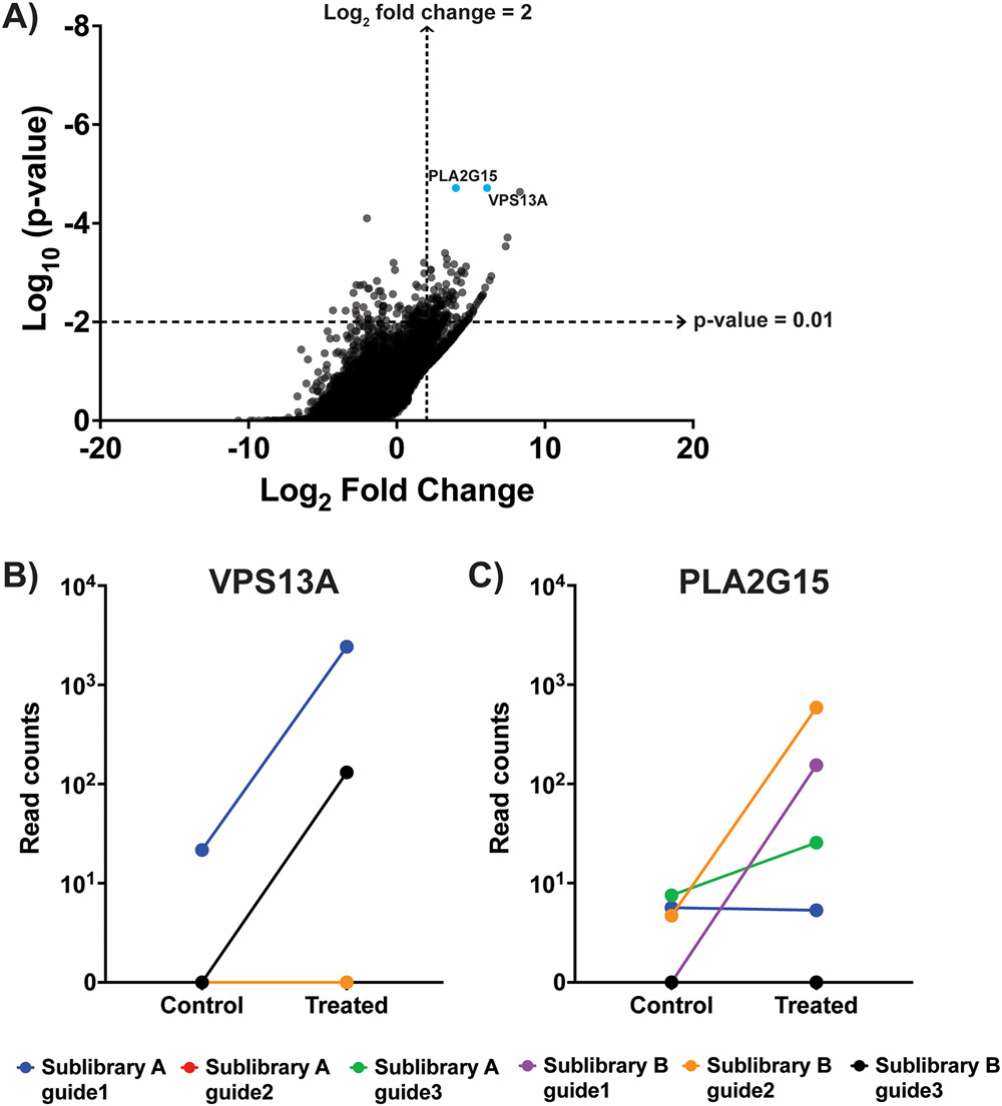
Significant hits from the CRISPR-Cas9 KO screen with βhc. (A) Results of the CRISPR-Cas9 knockout screen with βhc. The *x* axis represents the log fold change averaged for all guides against each gene in the treated population of cells versus the control population of cells. The two labeled genes, colored blue, are those considered significant based on a log fold change of ≥2, a *P* value of <0.01, ≥2 sgRNAs enriched in the βhc-treated versus control cell populations, and a false discovery rate of <20%. (B to C) Read counts of all the guides for the top 2 gene hits as measured in the βhc-treated and the control cell populations. An increase in the read count in the βhc-treated cells compared to that in the control cells indicates enrichment of that guide RNA through the course of the screening process.

The CRISPR-Cas9 screening method was repeated with group B Streptococcus βhc, to look for factors required for host cell death due to the action of the toxin, potentially including a toxin receptor. The concentration of βhc was measured in lytic units, with 1 lytic unit being the concentration causing 50% HeLa cell death (Fig. 3A). For the screen, HeLa cells were exposed to 3 lytic units of βhc, causing ≥90% cell death. Unlike the ILY screen, the percentage of cell death upon exposure to toxin did not decrease over the three rounds of the screen (Fig. 3B), suggesting that specific enrichment of resistant cell populations might not be occurring. Nevertheless, after the third round of intoxication, the top enriched sgRNA targets were tabulated, and the significant hits were selected as described above for the ILY screen. Based on our analysis strategy, only 2 of the top-ranked genes met the criteria for significance in βhc-mediated cell death (Fig. 4): VPS13A and PLA2G15.

VPS13A encodes a large protein called chorein, hypothesized to be involved in the transport and regulation of glycerophospholipids (31, 32). More recently, chorein has also been implicated as having a role in the endolysosomal pathway and in lysosomal degradation (33). PLA2G15 encodes a phospholipase localized to lysosomes and late endosomes, and it is known to catalyze the hydrolysis and degradation of cellular phospholipids (34, 35). Lysosomes and endosomes, in turn, play important roles in membrane repair (36, 37). Neither of the two gene targets are predicted to be cell surface proteins (and they are thus unlikely to act as receptors). However, both are involved with phospholipid homeostasis, which raises the possibility of VPS13A and PLA2G15 being involved in membrane repair and remodeling. This knowledge, combined with previous evidence showing that βhc activity *in vitro* can be curtailed by preincubation with phospholipids (17, 20), made VPS13A and PLA2G15 seem to be plausible players in βhc-mediated membrane pore formation. Thus, we decided to further explore the role of these two factors in βhc-mediated cell death.

### Validation of hits.

We next used the CRISPR-Cas9 approach to generate polyclonal knockout (KO) HeLa cells for the following selected genes: CD59 and PIGA from the ILY screen and VPS13A and PLA2G15 from the βhc screen. For each gene, one or two of the most enriched sgRNAs were packaged into lentivirus and transduced into HeLa cells. Positively transduced cells were selected using exposure to puromycin. Each KO cell population generated in this manner contained a mixture of mutations disrupting the respective gene and was therefore referred to as a polyclonal KO cell line. The same treatment was also repeated to create a control cell line using nontargeting (NT) sgRNAs.

**FIG 5:**
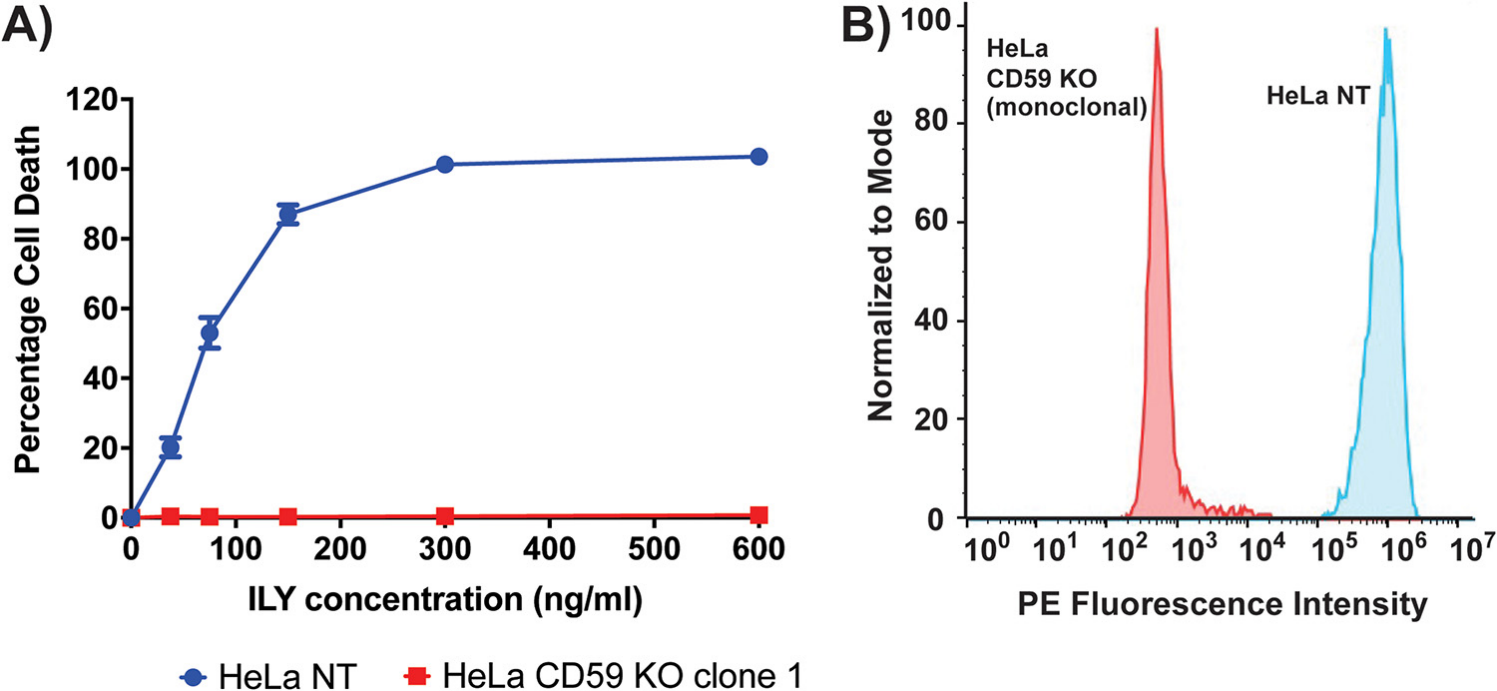
Selecting for a monoclonal HeLa CD59 KO cell line abrogates residual CD59 expression. (A) Percentages of cell death in HeLa NT (control cells, transduced with a nontargeting CRISPR sgRNA) and HeLa CD59 KO monoclonal cells when exposed to increasing concentrations of ILY, as measured by an LDH release cytotoxicity assay. The *P* value is <0.0001 for the points across all concentrations, as measured by two-way analysis of variance (ANOVA). The results shown are from one representative assay of >2 repeats. Each point is the mean value from 3 replicates, and error bars represent ±SD. Some error bars are contained within the point and therefore not visible. (B) Flow cytometry analysis of cell surface CD59 expression on HeLa wild type and HeLa CD59 monoclonal KO cells. The two-tailed *P* value is <0.0001 between the two populations, as measured by an unpaired *t* test. PE, phycoerythrin.

To test KO efficiency, polyclonal CD59 KO cells were exposed to ILY and the percentage of cell death measured using a lactate dehydrogenase (LDH) release assay. While polyclonal CD59 KO cells were more resistant to ILY toxicity than NT control cells, there was still ∼20% cell death at high concentrations of ILY, indicating that the polyclonal KO population still harbored cells susceptible to ILY, likely due to residual CD59 expression (Fig. S1A in the supplemental material). This hypothesis was further confirmed using flow cytometry for CD59 cell surface expression, which demonstrated a population of cells in the polyclonal KO cell line still expressing CD59 (Fig. S1B). To overcome this incomplete KO, limiting dilutions of the polyclonal CD59 KO cells were performed, in order to generate single-cell clones (i.e., monoclonal CD59 KO cell lines). Testing these monoclonal CD59 KO cells with ILY revealed clones that were completely resistant to the toxin; flow cytometry for CD59 expression also showed absence of CD59 expression in these cells (Fig. 5A and B). Similarly, polyclonal PIGA KO cells were exposed to ILY and limiting dilutions performed to generate monoclonal PIGA KO cell lines. Using exposure to ILY and the measurement of cell death by an LDH release assay, several clones were found to be completely resistant to the toxin (Fig. S2).

**FIG 6:**
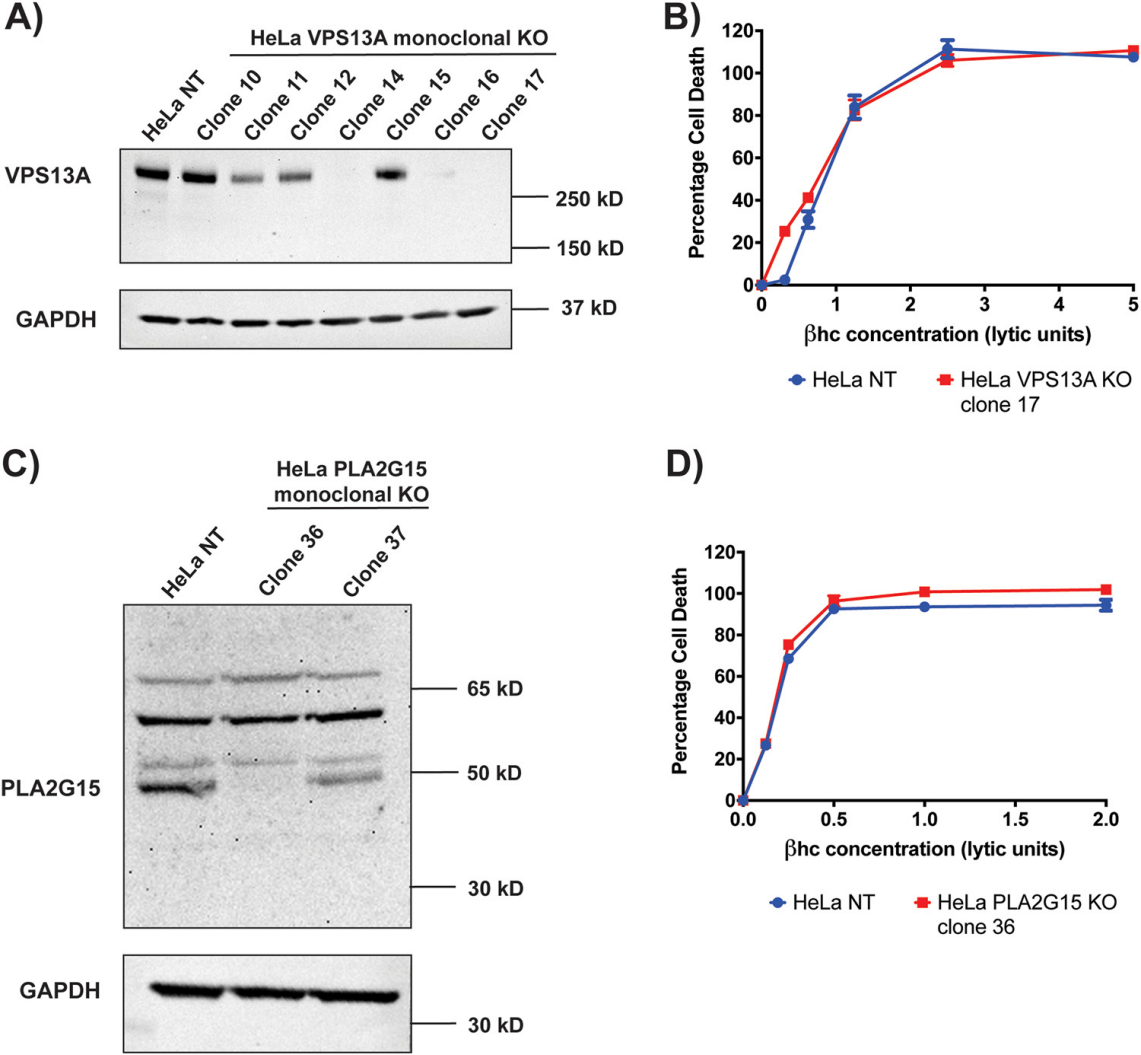
Knocking out VPS13A or PLA2G15 has no effect on susceptibility to βhc. (A) Western blot analysis of VPS13A (∼360 kDa) and GAPDH (∼36 kDa) expression in whole-cell lysates of HeLa control cells and HeLa VPS13A monoclonal KO cells. The cell lines showing complete abrogation of VPS13A expression were tested for resistance to βhc. (B) A representative LDH cytotoxicity assay with HeLa VPS13A KO clone 17, demonstrating no effect of knocking out VPS13A on susceptibility to βhc. The results shown are from one representative assay of >2 repeats. Each point is the mean value from 3 replicates, and error bars represent ±SD. Some error bars are contained within the point and therefore not visible. (C) Western blot analysis of PLA2G15 (∼43 kDa) and GAPDH (∼36 kDa) expression in whole-cell lysates of HeLa control cells and HeLa PLA2G15 monoclonal KO cells. The cell line showing complete abrogation of PLA2G15 expression (clone 36) was tested for resistance to βhc. (D) An LDH cytotoxicity assay with HeLa PLA2G15 KO clone 36, demonstrating no effect of knocking out PLA2G15 on susceptibility to βhc. The results shown are from one representative assay of >2 repeats. Each point is the mean value from 3 replicates, and error bars represent ±SD. Some error bars are contained within the point and therefore not visible.

Based on these results, monoclonal VPS13A KO cells were generated as described above. The KO efficiency of the polyclonal VPS13A KO cell line, as well as several monoclonal VPS13A KO cell lines, was investigated using western blotting (Fig. 6A and Fig. S3). For the monoclonal cell lines shown to have complete knockout of VPS13A, sensitivity to βhc was analyzed using LDH release cell death assays (Fig. 6B). Similarly, monoclonal PLA2G15 KO cells were generated and the KO efficiency investigated using western blotting (Fig. 6C). The sensitivity of a monoclonal PLA2G15 KO cell line to βhc was analyzed using LDH release assays (Fig. 6D). There was no effect from knocking out either VPS13A or PLA2G15 on βhc-mediated cell death.

## DISCUSSION

The CRISPR-Cas9 system has proven to be an important laboratory tool for targeted disruption of genes in human cells. CRISPR-Cas9 knockout (or loss-of-function) screens, in particular, have been invaluable in recent years, with a growing number of studies using them to find novel host cell targets required by various bacterial toxins involved in pathogenesis. A CRISPR-Cas9 knockout screen with Escherichia coli Shiga toxin (Stx) identified two genes of previously unknown function (TM9SF2 and LAPTM4A) as being important for Stx toxicity, and further experiments confirmed that the two genes played a role in biosynthesis of the known Stx receptor Gb3 (25). Similarly, a CRISPR-Cas9 knockout screen conducted with Staphylococcus aureus alpha-toxin identified genes regulating the expression of the known alpha-toxin receptor ADAM10 and also uncovered a possible role of lipid raft formation in alpha-toxin cytotoxicity (22). To better understand the mechanism of action of the group B Streptococcus (GBS) pore-forming toxin (PFT) β-hemolysin/cytolysin (βhc), we screened for genes required for βhc toxicity in HeLa cells by employing CRISPR-Cas9 knockout screening technology.

As a control, we conducted a screen using the Streptococcus intermedius pore-forming toxin intermedilysin (ILY), which requires the GPI-anchored protein human CD59 as a host cell surface receptor (26). As expected, the ILY control screen identified CD59 as the major target for ILY-mediated cytotoxicity. Additionally, the ILY screen also identified multiple genes in the biosynthesis pathway for the GPI anchor, which is required for cell surface localization of CD59. We validated the importance of CD59 as the top target for ILY via the production of a targeted monoclonal KO cell line, which proved to be fully resistant to the toxin. Of note, a recent study reported a CRISPR-Cas9 knockout screen with ILY that confirmed CD59 as the major factor but also identified target cell heparan sulfates as modulators of ILY activity (27). Our ILY screen did not identify heparan sulfates. A possible reason is that the prior study used substantially lower doses of ILY for the screen and noted that heparan sulfates disappeared as targets when higher ILY concentrations were used, whereas we used a comparatively high concentration of ILY, causing ≥90% lysis in our screen. This difference likely accounts for our screen identifying only CD59 and GPI synthesis pathway components as targets.

The βhc screen was also conducted with a high concentration of toxin, causing ≥90% lysis, to ensure stringent selection of resistant cells. The screen identified two potential targets: VPS13A and PLA2G15. Notably, both targets had higher FDRs and lower sgRNA enrichment values than the ILY screen targets, marking them as potentially less robust hits. However, since the combination of FDR, *P* value, number of enriched guides, and log fold change for the targets was within our range of criteria for significance, they merited a closer look. We created monoclonal knockout cell lines for each of these genes. However, upon exposure to βhc, neither of the two cell lines were resistant to the toxin. These results indicate that while CRISPR-Cas9 knockout screens are valuable tools in determining host cell targets for bacterial toxins, this strategy has limitations that need to be taken into consideration.

CRISPR-Cas9 KO screens cannot identify targets that encode functions essential for cellular survival. Therefore, even in the setting of our findings, it remains possible that βhc-induced cell death may require one or more essential host genes. βhc lyses eukaryotic cells from a wide variety of host species (17), indicating that it may require targets that are highly conserved and essential to cell survival. Previous data have shown that preincubation with purified phospholipids inhibits βhc toxicity (17, 20), indicating that interaction with phospholipids might be important for the βhc mechanism of action. This finding is consistent with the known affinity of polyenes for cell membrane sterols and phospholipids and with the fact that the formation of polyene-lipid complexes can lead to cytolysis (38–40). However, phospholipids are a major constituent of the cell membrane lipid bilayer, so it is possible that genes that modulate phospholipid expression in the cell membrane may be essential. Furthermore, the strategy of knocking out a single gene in each cell may fail to identify targets with substantial functional redundancy. This is evident in published data about other toxins, such as the Bacillus thuringiensis toxin Cry1AC, for which two host ATP-binding cassette transporter genes need to be knocked out together to see any effect on Cry1AC toxicity (41). If multiple host genes have interchangeable functions in facilitating βhc toxicity and disrupting one gene alone does not decrease susceptibility to βhc, neither would be identified by the CRISPR-Cas9 knockout screen. Finally, the high dose of βhc used in our screen (causing ≥90% cell death) could potentially be masking targets that might appear only with lower doses of the toxin, as was demonstrated by the previously published screen results for ILY (27). Further experimentation would therefore be needed to confirm the requirements for intoxication by sublytic concentrations of βhc.

The βhc molecule is composed of a hydrophobic polyene chain, flanked by polar head groups (11). Recent data suggest that the length of the polyene chain and the polarity of the βhc molecule are important for toxicity (21). Decreasing the chain length to less than the thickness of a phospholipid bilayer decreases the lytic ability of βhc, and this effect can be partially overcome by changing one of the head groups to a hydrophobic moiety (21). These results point to the possibility that, unlike traditional PFT, βhc might not require binding to a specific receptor on the host cell surface, instead perturbing a host cell membrane in a nonspecific manner because of hydrophobic interactions. Known examples of such behavior include the pore-forming peptides alamethicin and gramicidin A (42).

In summary, our CRISPR-Cas9 knockout screen did not identify any host genes essential for βhc function that could be validated, and possible reasons include pathway redundancy and absence of a nonessential receptor for βhc. However, our study was limited to one cell line and one screening strategy. Additional experiments in different cell lines and with different loss-of-function screening tools are likely needed to definitively conclude whether βhc requires host factors for effective cell intoxication.

## MATERIALS AND METHODS

### Cell culture.

Lenti-X 293T cells were cultivated in Dulbecco’s modified Eagle medium (DMEM) supplemented with 10% heat-inactivated fetal bovine serum and penicillin/streptomycin. HeLa cells were cultivated in Eagle minimum essential medium (EMEM) supplemented with 10% heat-inactivated fetal bovine serum (HI-FBS) and penicillin/streptomycin. Cells were grown in humidified incubators at 37°C and 5% CO_2_. For transduced cells, the medium was further supplemented with 0.9 μg/ml puromycin.

### Toxins.

Recombinant ILY was prepared as described previously (43). Briefly, the gene encoding ILY was cloned into pET28a and the plasmid transformed into E. coli T7Iq. Protein expression was induced by the addition of IPTG (isopropyl-β-d-thiogalactopyranoside). Cells were lysed with a lysis buffer (50 mM NaH_2_PO_4_, 300 mM NaCl, 10 mM imidazole). ILY was purified from the lysate, using HisTrap columns with a fast protein liquid chromatography (FPLC) unit, into elution buffer (50 mM NaH_2_PO_4_, 300 mM NaCl, 250 mM imidazole) and then buffer exchanged into phosphate-buffered saline (PBS) using Amicon ultra-4 filters. ILY purity was assessed via gel electrophoresis on a NuPAGE Bis-Tris gel, and the concentration was measured using the Bradford assay.

βhc was prepared as described previously (44). Briefly, Streptococcus agalactiae strain CNCTC10/84 Δ*covR* was grown overnight (stationary growth at 37°C), and bacterial cells collected by centrifugation. The bacterial pellet was resuspended (2% soluble starch plus 1% d-glucose in PBS) and incubated at 37°C for 2 h. The suspension was centrifuged, and the supernatant mixed with ice-cold methanol in a 1:1 ratio and stored overnight at −20°C to precipitate starch-bound βhc. The mixture was centrifuged to obtain the βhc pellet, which was resuspended in PBS. The same procedure was repeated with Streptococcus agalactiae strain CNCTC10/84 Δ*cylE*, which does not produce βhc, resulting in Δ*cylE* extract to be used as a control for all experiments with βhc.

### Cell viability assays.

The CellTiter-Glo assay for cell viability was conducted according to the manufacturer’s instructions. Briefly, HeLa cells were plated in a 96-well plate in EMEM supplemented with 10% HI-FBS and penicillin/streptomycin. On the next day, the medium was aspirated from the wells and cells were incubated in appropriate dilutions of toxin in fresh medium for 1.5 h in a humidified incubator at 37°C and 5% CO_2_. Then, the plates were incubated at room temperature (RT) for 30 min before the addition of the CellTiter-Glo reagent directly to the medium in the wells at a 1:1 ratio. Cells were allowed to lyse for 2 min on a shaker and then for 10 min under stationary conditions at RT. CellTiter-Glo luminescence was recorded using a plate reader. Cell viability was calculated relative to the results for control wells with 0% cell death (cells incubated in 1× PBS) and 100% cell death (cells incubated in 0.2% Triton X-100). For each concentration of toxin in every assay, three replicate wells were prepared, with the mean and standard deviation (SD) calculated based on those measurements.

The LDH cytotoxicity assay was also conducted according to the manufacturer’s instructions. Briefly, 2.0 × 10^4^ HeLa cells were seeded per well in a 96-well plate in EMEM supplemented with 10% HI-FBS and penicillin/streptomycin. The next day, the medium was aspirated from the wells and cells were incubated in appropriate dilutions of toxin in fresh medium for 2 h in a humidified incubator at 37°C and 5% CO_2_. After incubation, the plates were centrifuged to separate cellular debris, and 100 μl of the supernatant transferred to a new 96-well plate. To these wells, 100 μl of reaction mixture consisting of dye solution and catalyst (prepared according to the manufacturer’s protocol) was added, and the plates incubated in the dark for 30 min. The absorbance of samples was measured at 492 nm.

### Production of lentiviral vector library.

Lenti-X 293T cells were seeded in T225 flasks at 3.5 × 10^6^ cells per flask. The next day, each flask of cells was cotransfected with the lentiviral packaging vectors pMD2.G (15.3 μg) (plasmid number 12259; Addgene) and psPAX2 (23.4 μg) (plasmid number 12260; Addgene), and the GeCKO LentiCRISPRv2 plasmid library (30.6 μg) (gifted by Feng Zhang; plasmid number 1000000048; Addgene) (29), using Lipofectamine 2000 and plus reagent, as outlined previously (45). After 4 h, the medium was replaced with fresh prewarmed DMEM. Forty-eight h later, the lentivirus-containing supernatant was collected and cellular debris filtered using a 0.45-μm filters. The lentivirus was aliquoted and stored at −80°C.

### Genome-wide CRISPR-Cas9 knockout screen in HeLa cells.

The CRISPR screen was conducted based on a published protocol (45). Briefly, cells were transduced with the GeCKO v2 viral library using the spinfection method. To titrate for optimal virus volumes needed for achieving an MOI of 0.3, HeLa cells were plated in 24-well plates. Twenty-four hours later, medium in the wells was replaced with fresh EMEM supplemented with 3% FBS and 8 μg/ml Polybrene and virus dilutions made in PBS were added to the wells. The plates were centrifuged at 1,000 × *g* for 2 h at 37°C before being left in a humidified incubator (37°C, 5% CO_2_) for 24 h. Cells were detached with TrypLE, counted, and replated into wells of a 96-well plate (2 wells with 0.7 μg/ml puromycin, 2 wells without puromycin). The cells were incubated at 37°C for 96 h, cell viability quantified using CellTiter-Glo, and the MOI for each virus condition calculated as described previously (45). The virus volume yielding an MOI of 0.3 was chosen for the large-scale screens.

The GeCKO v2 sgRNA library is divided into two sublibraries (sublibrary A and sublibrary B), with each sublibrary constituting 3 sgRNAs per gene, for a total of 6 sgRNAs per gene in the combined library. The starting number of cells was determined to be at least 500 times the number of total guides in the library (500× coverage; ∼3.3 × 10^7^ cells for each GeCKO v2 sublibrary). The cells were plated in 6-well plates, and spinfection was conducted as outlined above. Twenty-four hours after spinfection, cells were detached using TrypLE, pooled, and replated in T225 flasks in fresh medium supplemented with 0.9 μg/ml puromycin for 7 days.

To conduct each screen, GeCKO v2 library cells were plated in T225 flasks, maintaining 500× coverage. Toxin was added at the appropriate concentration (100 ng/ml for ILY; 3 lytic units for βhc), and the cells were incubated at 37°C for 2 h. After toxin treatment, cells were washed with PBS, detached using TrypLE, counted, and replated in fresh medium. The cells were allowed to recover and grow under puromycin selection for approximately 2 weeks, until they had proliferated in number to at least ∼3.3 × 10^7^ (the required number for maintaining 500× representation per CRISPR sublibrary), before the next round of toxin exposure.

Following three rounds of toxin exposure, genomic DNA from the surviving cells was extracted using the Qiagen blood and cell culture DNA midikit. The sgRNA regions were amplified and barcoded using nested PCR, as outlined previously (45). Equimolar ratios of the nested PCR products were pooled and gel extracted using the QIAquick gel extraction kit. The concentrations of the gel-extracted products were measured using a chip-based capillary electrophoresis machine and sequenced by NYU Langone’s Genome Technology Center on the Illumina NextSeq 500 platform. Sequence data were analyzed using MAGeCK (version 0.5.9), and the CRISPR screen data were processed as described previously (30, 46).

### Generation of knockout cell lines.

For the two most enriched sgRNAs of each relevant gene target, oligonucleotides (plus strand and minus strand) were designed and ordered from IDT, as outlined previously (45). These were phosphorylated and annealed using T4 polynucleotide kinase. An empty pLentiCRISPR plasmid backbone (gifted by Feng Zhang; Addgene plasmid number 52961) (29) was digested using FastDigest Esp3I and gel extracted with the QIAquick gel extraction kit. The annealed oligonucleotides were ligated into the digested pLentiCRISPR using T4 DNA ligase. The ligated plasmids were transformed into NEB 5alpha cells and ligation confirmed via sequencing.

The correctly sequenced plasmids were packaged in lentivirus as outlined above, with modifications. Lenti-X 293T cells were seeded in poly-d-lysine coated 6-well plates and cotransfected using Opti-MEM and XtremeGene 9 DNA transfection reagent as follows. One microgram of the correctly sequenced plasmid was mixed with lentiviral packaging vectors pCMV-Gag-Pol and pCMV-VSVg (gifted by Charles Rice) in a ratio of 1:0.8:0.2. A medium master mixture was prepared with 100 μl Opti-MEM and 6 μl XtremeGene 9 for each well, mixed together with the total plasmid DNA, and added to each well. Lentivirus-containing supernatant was collected at 48 h and 72 h posttransfection. Sterile HEPES and Polybrene were added to the lentivirus at final concentrations of 20 mM and 4 μg/ml, respectively. The lentivirus was aliquoted and stored at −80°C.

To transduce HeLa cells with lentivirus, HeLa cells were seeded in 6-well plates. Once the cells reached confluence, the medium in the wells was replaced with fresh EMEM supplemented with 3% FBS and 8 μg/ml Polybrene. To each well, 300 μl of relevant lentivirus was added. Spinfection was carried out as outlined above. Twenty-four hours later, cells were replated into T75 cell culture flasks and selected in 0.9 μg/ml puromycin for 7 days. To generate single-cell clones from polyclonal KO cell lines, limiting dilutions were performed in 96-well plates and the growth of isolated clones assessed under a microscope until the cells were sufficiently grown.

### Western blotting.

To prepare HeLa cell lysates for western blotting, 5 × 10^6^ cells of each relevant cell line were resuspended in ice-cold radioimmunoprecipitation assay (RIPA) buffer and constant agitation was maintained at 4°C for 30 min. Centrifugation was then carried out at 4°C for 20 min at 13,400 × *g*. The lysate was transferred to a fresh tube and stored at −20°C. Cell lysates were mixed with loading dye and reducing agent and loaded on a NuPAGE 4-to-12% Bis-Tris gel. The proteins from the gel were transferred to a polyvinylidene difluoride (PVDF) membrane, blocked with 5% nonfat dry milk in Tris-buffered saline (TBS) plus 1% Tween 20, and incubated with primary antibody. For anti-PLA2G15 antibody (sc-376078; Santa Cruz Biotechnology), incubation was carried out overnight at 4°C at an antibody dilution of 1:200. For anti-VPS13A antibody (NBP1-85641; Novus Biologicals), incubation was carried out for 1 h at RT at an antibody dilution of 1:400. After primary antibody incubations, membranes were washed in TBS plus 1% Tween 20 and incubated in secondary antibody (goat anti-rabbit IgG [31460; Thermo Fisher] at 1:5,000 dilution or goat anti-mouse IgG [31430; Thermo Fisher] at 1:10,000 dilution) for 1 h at RT. All primary and secondary antibodies were diluted in 5% nonfat dry milk in TBS plus 1% Tween 20. Membranes were imaged on an iBright CL1000 using Pierce ECL western blotting substrate. Glyceraldehyde-3-phosphate dehydrogenase (GAPDH) was used as a loading control, with anti-GAPDH antibody (sc-32233; Santa Cruz Biotechnology) at 1:200 dilution.

### Flow cytometry.

Flow cytometry to visualize CD59 expression was carried out using indirect staining. Briefly, 2.5 × 10^6^ cells were washed and resuspended in 500 μl of ice-cold PBS plus 10% FBS plus 1% sodium azide. Anti-CD59 (MEM-43) mouse IgG2a primary antibody (sc-51565; Santa Cruz Biotechnology) was added to the cell suspension at a dilution of 1:20, and cells were incubated for 30 min at RT in the dark. After incubation, cells were washed with ice-cold PBS and resuspended in goat anti-mouse IgG2a Alexa Fluor 647 secondary antibody (A21241; Thermo Scientific) diluted 1:400 in PBS plus 3% bovine serum albumin (BSA). Cells were incubated again for 30 min at RT in the dark. After this incubation, cells were washed and resuspended in ice-cold PBS plus 3% BSA plus 1% sodium azide and filtered using a 40-μm cell filter. Stained cells were analyzed on a CytoFlex analyzer.

### Data availability.

Sequences are available in the NCBI Sequence Read Archive under BioProject accession number PRJNA794185.
