# Supplementary material for: Genome-Wide CRISPR-Cas9 Screen Does Not Identify Host Factors Modulating Streptococcus agalactiae β-Hemolysin/Cytolysin-Induced Cell Death
Source: Microbiol Spectr. 2022 Feb 2;10(1):e02186-21. doi: 10.1128/spectrum.02186-21 (PMC8865549; doi:10.1128/spectrum.02186-21)
Supplement: SUPPLEMENTAL FILE 1 — Supplemental material. Download SPECTRUM02186-21_Supp_1_seq3.pdf, PDF file, 0.5 MB [file spectrum02186-21_supp_1_seq3.pdf]

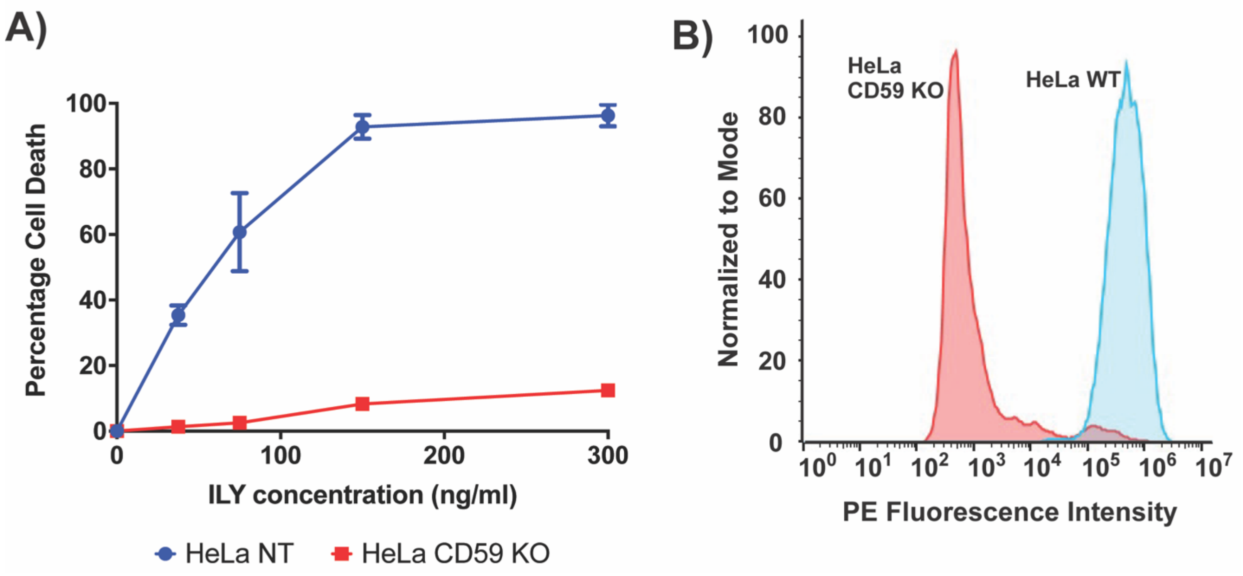

Fig S1: Residual CD59 expression in HeLa CD59 polyclonal KO cells.

(**A**) Percentage cell death in HeLa NT (control cells, transduced with a non-targeting CRISPR sgRNA) and HeLa CD59 KO polyclonal cells when exposed to increasing concentrations of ILY, as measured by an LDH-release cytotoxicity assay. The p-value is <0.0001 for the points across all concentrations, as measured by a two-way ANOVA. Figure shows one representative assay of several repeats. Each point is the mean of 3 replicates, and error bars represent ±SD. Some error bars are contained within the point and therefore not visible.

(**B**) Flow cytometry analysis of cell surface CD59 expression HeLa WT and HeLa CD59 polyclonal KO cells. Histogram shows fluorescence intensity of the antibody in the two cell populations. The small overlap of the HeLa CD59 KO cells peak (red) with the HeLa WT cells (blue) indicates residual CD59 cell surface expression in a subset of the HeLa CD59 polyclonal KO cell population. The two-tailed p-value is <0.0001 between the two populations, as measured by an unpaired t-test.

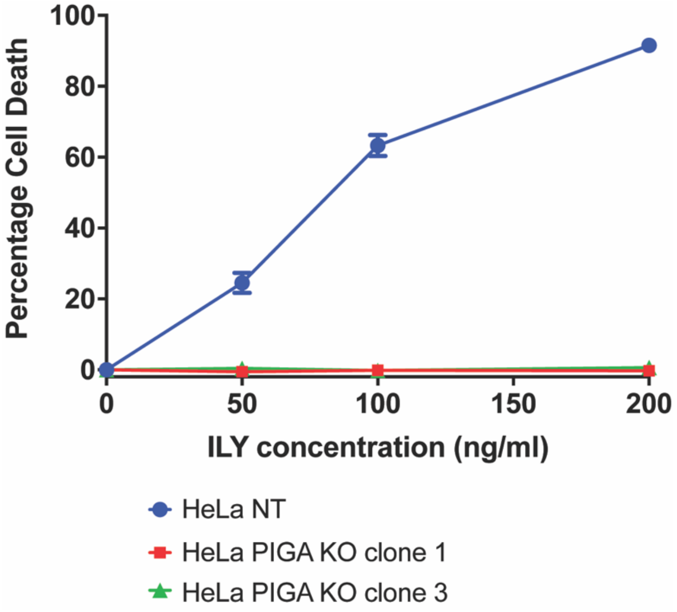

Fig S2: Monoclonal HeLa PIGA KO cell lines are resistant to ILY Percentage cell death in HeLa NT (control cells, transduced with a non-targeting CRISPR sgRNA) and HeLa PIGA KO monoclonal cell lines when exposed to increasing concentrations of ILY, as measured by an LDH-release cytotoxicity assay. The p-value is <0.0001 for the points across all concentrations between HeLa NT and HeLa PIGA KO clone 1, and between HeLa NT and HeLa PIGA KO clone 3 (as measured by two-way ANOVA). Figure shows one representative assay of several repeats. Each point is the mean of 3 replicates, and error bars represent ±SD. Some error bars are contained within the point and therefore not visible.

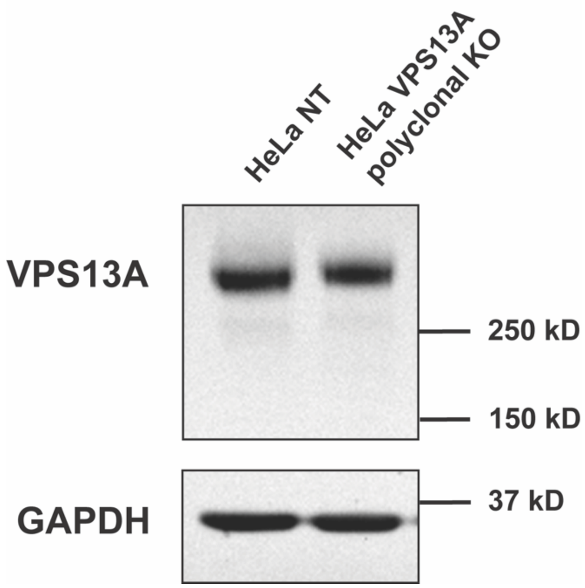

Fig S3: Residual expression of VPS13A in HeLa VPS13A polyclonal KO cells Western blot analysis of VPS13A (~360 kDa) and GAPDH (~36 kDa) expression in whole cell lysates of HeLa control cells and HeLa VPS13A polyclonal KO cells.
